# Supplementary material for: Raman microscopy of cryofixed biological specimens for high-resolution and high-sensitivity chemical imaging
Source: Sci Adv. 2024 Dec 11;10(50):eadn0110. doi: 10.1126/sciadv.adn0110 (PMC11633761; doi:10.1126/sciadv.adn0110)
Supplement: Supplementary file 1 — Figs. S1 to S10 Tables S1 and S2 References [file sciadv.adn0110_sm.pdf]

Science Advances
AAAS

Supplementary Materials for

Raman microscopy of cryofixed biological specimens for high-resolution and high-sensitivity chemical imaging

Kenta Mizushima *et al.*

Corresponding author: Katsumasa Fujita, fujita@ap.eng.osaka-u.ac.jp

Sci. Adv. **10**, eadn0110 (2024)
DOI: 10.1126/sciadv.adn0110

This PDF file includes:

Figs. S1 to S10
Tables S1 and S2
References

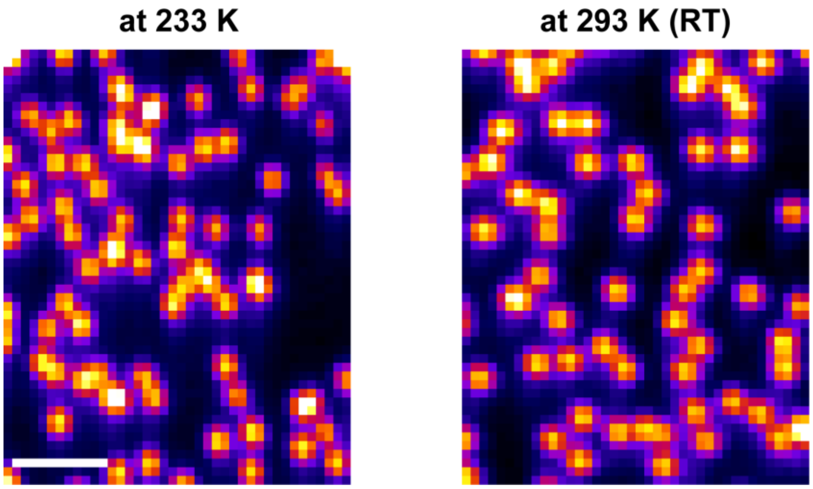

Fig. S1.

Fluorescence images of fluorescence beads with a diameter of 0.2 µm (F8809, Invitrogen). (Left) The beads measured at 233 K in the cryostat with liquid nitrogen circulation. (Right) The beads measured at 293 K without liquid nitrogen circulation. Scale bar: 2 µm. The fluorescence images were acquired using the slit-scanning Raman microscope described in Materials and Methods. The exposure time was 10 ms/line. The interval between acquiring neighboring lines, equivalent to the detector readout time, was 200 ms. The scanning pitch was 90 nm. The fluorescence images were reconstructed from zeroth-order light diffracted by the spectrophotometer. The slit width of spectrophotometer was 50 µm (~1.0 A.U. confocal slit).

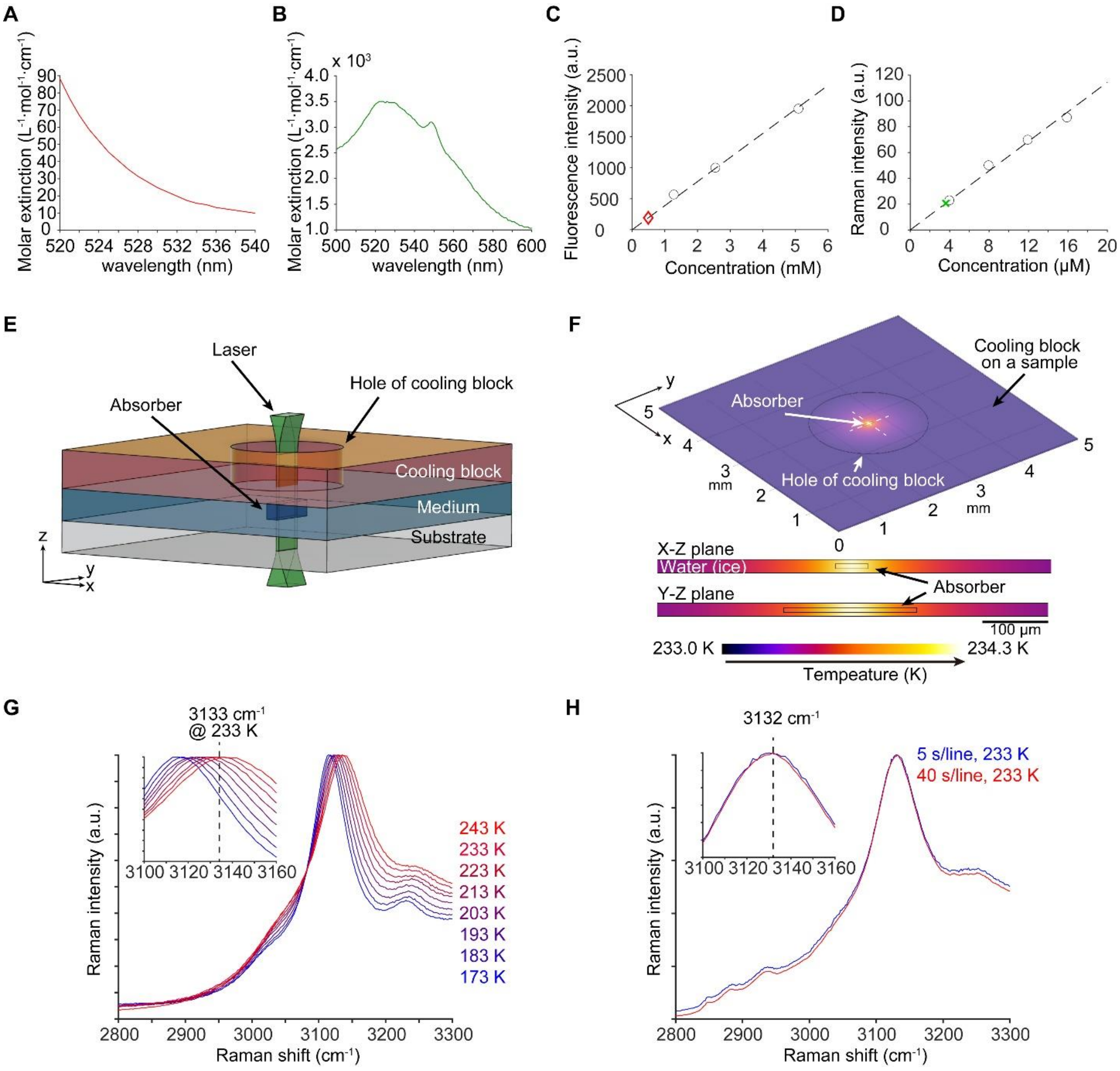

Fig. S2.

Local heating by laser irradiation in Raman imaging of cell samples. (**A**) Molar extinction coefficient of FAD calculated from the excitation spectrum taken by a spectrophotometer. (**B**) Molar extinction coefficient of reduced cytochrome *c* calculated from the excitation spectrum taken by a spectrophotometer. (**C**) Plots of the fluorescence intensity to the concentration for FAD in aqueous solutions (black circle), a linear calibration curve fit to the plots (black dashed line), and the plot of intracellular autofluorescence intensity to the concentration estimated from the fitting curve (red diamond). (**D**) Plots of the Raman intensity to the concentration for reduced cytochrome c in aqueous solutions (black circle), a linear calibration curve fit to the plots (black dashed line), and the plot of intracellular Raman intensity to the concentration estimated from the fitting curve (green cross). FAD and reduced cytochrome *c* in water with each concentration was measured by our Raman microscope for obtaining the calibration curves. Intracellular concentration of FAD and reduced cytochrome *c* was obtained from the calibration curves and

fluorescence and Raman intensity of HeLa cells. (**E**) A schematic of the 3D model for simulation of local heating with laser irradiation by COMSOL. Aspect ratio of each component is modified for visibility. The actual parameters used in the calculation were listed in the Table **S1**. (**F**) Distribution of the sample temperature after laser irradiation for 70 s (upper). Enlarged images of x-z and y-z cross-sections along with the white lines in the x-y plot are also shown (lower). Initial temperature of the sample was 233 K and temperature of the cooling block was kept in 233 K during the simulation. (**G**) Raman spectra of water measured at low temperature (173 to 243 K) with an exposure time of 10 s/line. (**H**) Raman spectra of cryofixed HeLa cells at 233 K measured with exposure times of 5 s/line (blue line) and 40 s/line (red line). The laser power was 3.0 $mW/\mu m^2$ for Fig. **S2G**-**H**.

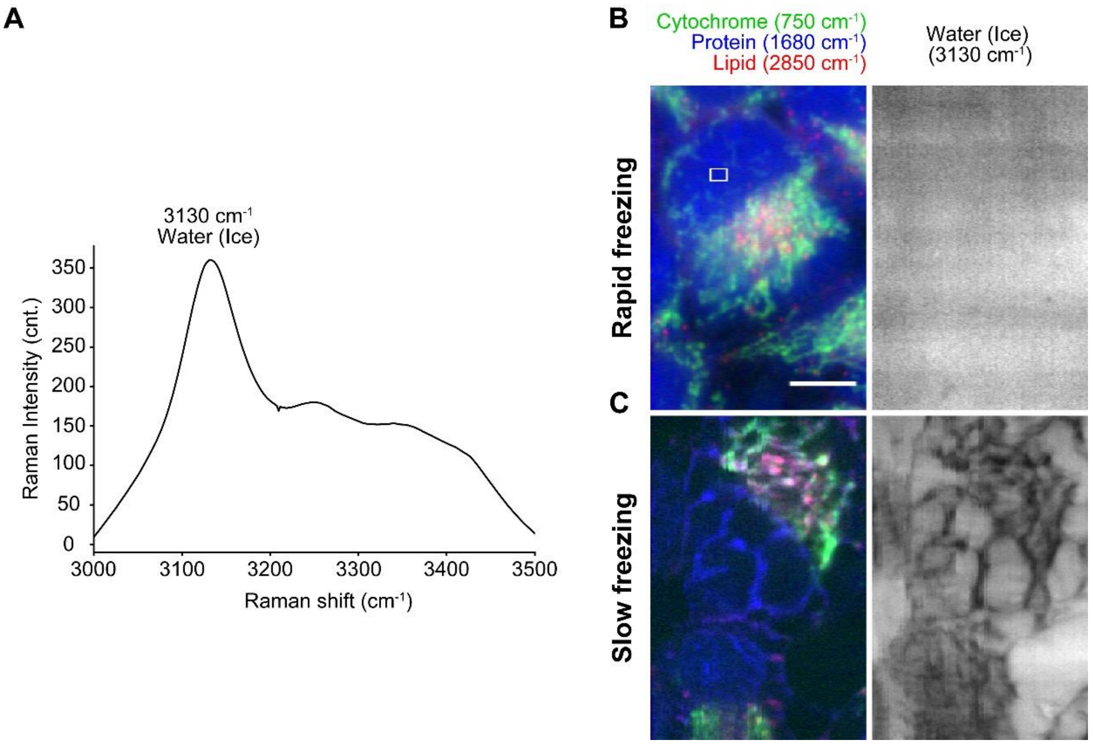

Fig. S3.

Raman measurement of ice crystal formation in HeLa cell measurements. (**A**) The Raman spectrum averaged over the white square in (**B**). (**B**) Raman images presenting the distributions of cytochrome, protein, and lipid in HeLa cells (left) and ice crystals over the imaging area (right), with rapid freezing by liquid propane at 88 K. (**C**) Raman images presenting the distributions of cytochrome, protein, and lipid in HeLa cells (left) and ice crystals over the imaging area (right) with slow freezing at the cooling rate of 1 K/min using the metal plate in the custom cryostat. Exposure time was 5 s/line. Scale bar: 10 µm.

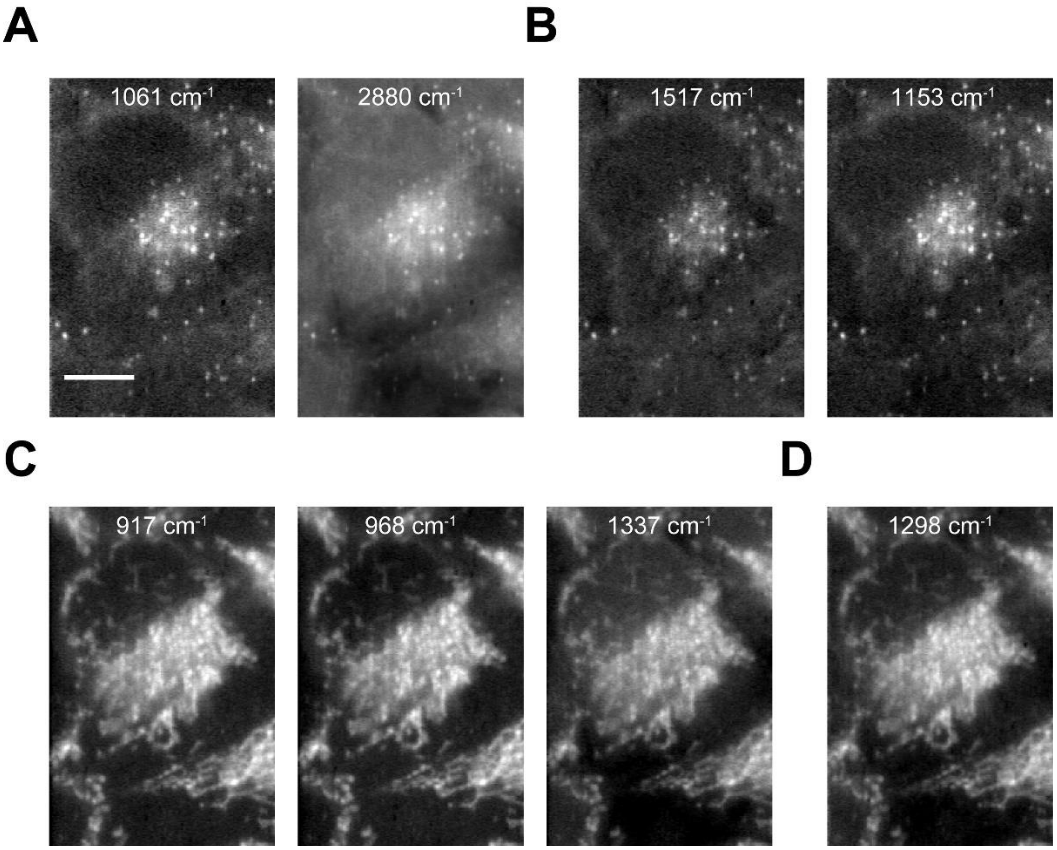

Fig. S4.

Raman images of cryofixed HeLa cells reconstructed from Raman bands that appeared or increased only in cryofixed condition. (**A**) The images reconstructed at 1061 and 2880 cm^{-1} (lipid) (**B**) The images reconstructed at 1151 and 1517 cm^{-1} (carotenoids). (**C**) The images reconstructed from 917, 968, and 1337 cm^{-1} (cytochromes). (**D**) The image reconstructed at 1298 cm^{-1} (cytochromes and lipid). Exposure time: 5 s/line. Scale bar: 10 µm.

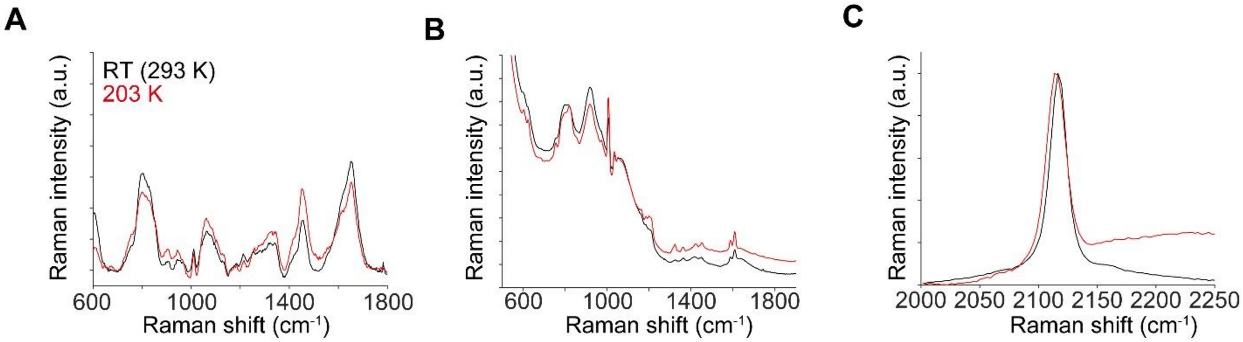

Fig. S5. Temperature dependence of Raman spectra of biological molecules. Raman spectra of (**A**) 1 mM bovine serum albumin, (**B**) 1 mM phenylalanine, and (**C**) 20 mM EdU aqueous solutions. The exposure time and the laser power were 60 s and 3.0 mW/μm^2, respectively.

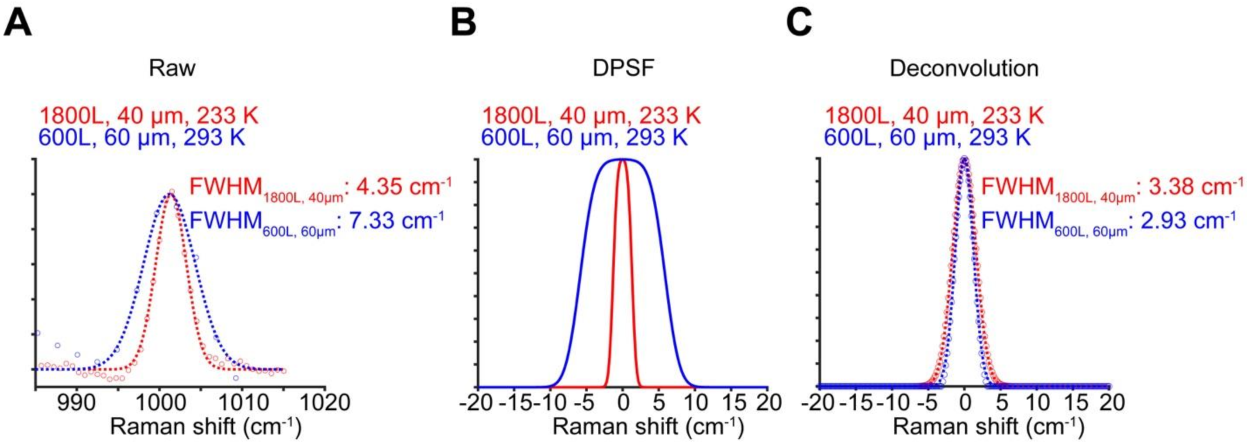

Fig. S6.

Estimation of Raman band width of phenylalanine at 1001 cm^{-1}. (A) Raman spectra of HeLa cells obtained at a high spectral resolution with a 40 µm slit width (~0.8 Airy Unit confocal slit) and 1800 L/mm grating at cryofixed condition (red) and a low spectral resolution with a 60 µm slit width (~1.2 Airy Unit confocal slit) and 600 L/mm grating at room temperature (blue). Red and blue solid lines are fitted using Gaussian functions. **(B)** Detection point spread functions (DPSF) of the spectrophotometer used in the Raman microscope for each condition, which were estimated by convolution of the slit width and the point spread function of the system measured using the neon spectrum using the slit width (10µm) smaller than the pixel size. **(C)** Raman spectra of HeLa cells estimated by deconvolving the measured spectrum (A) with DPFS (B). Note that the Gaussian functions were used for the deconvolution.

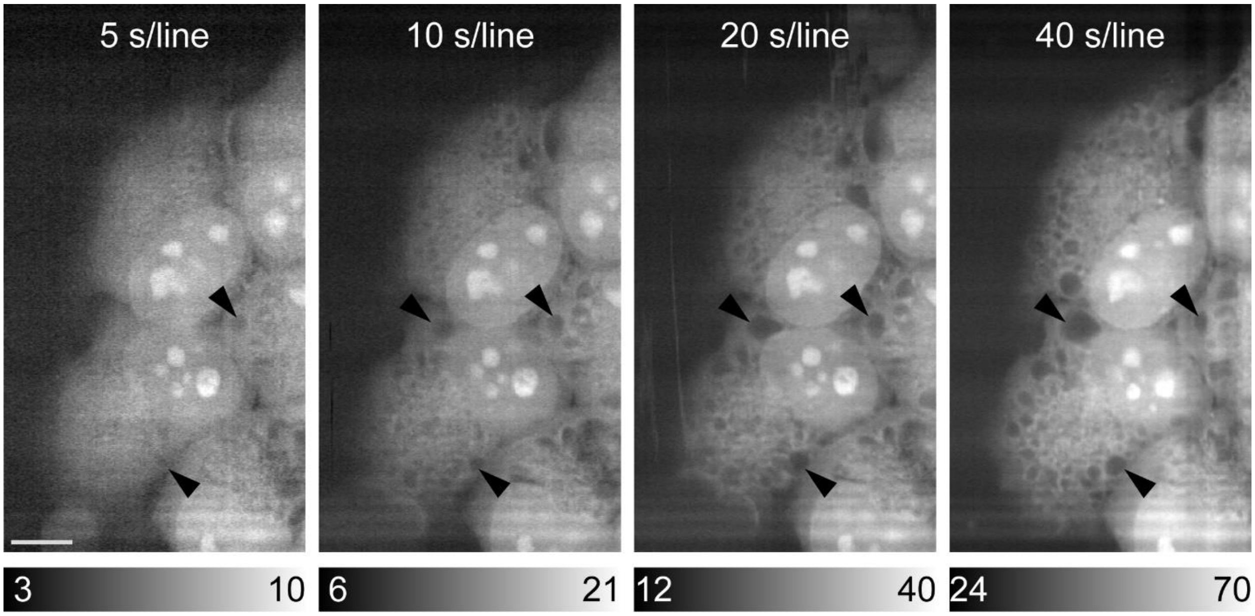

Fig. S7.

Sample modification during repeated Raman imaging acquisitions of HeLa cells fixed by paraformaldehyde. The images were reconstructed by the intensity at 1680 cm^{-1} assigned to the amide-I vibrational mode. Black arrows indicated the modified position during Raman imaging. The exposure times were set to 5, 10, 20, and 40 s/line for the first, second, third, and fourth acquisitions, respectively. Scale bar: 10 μm.

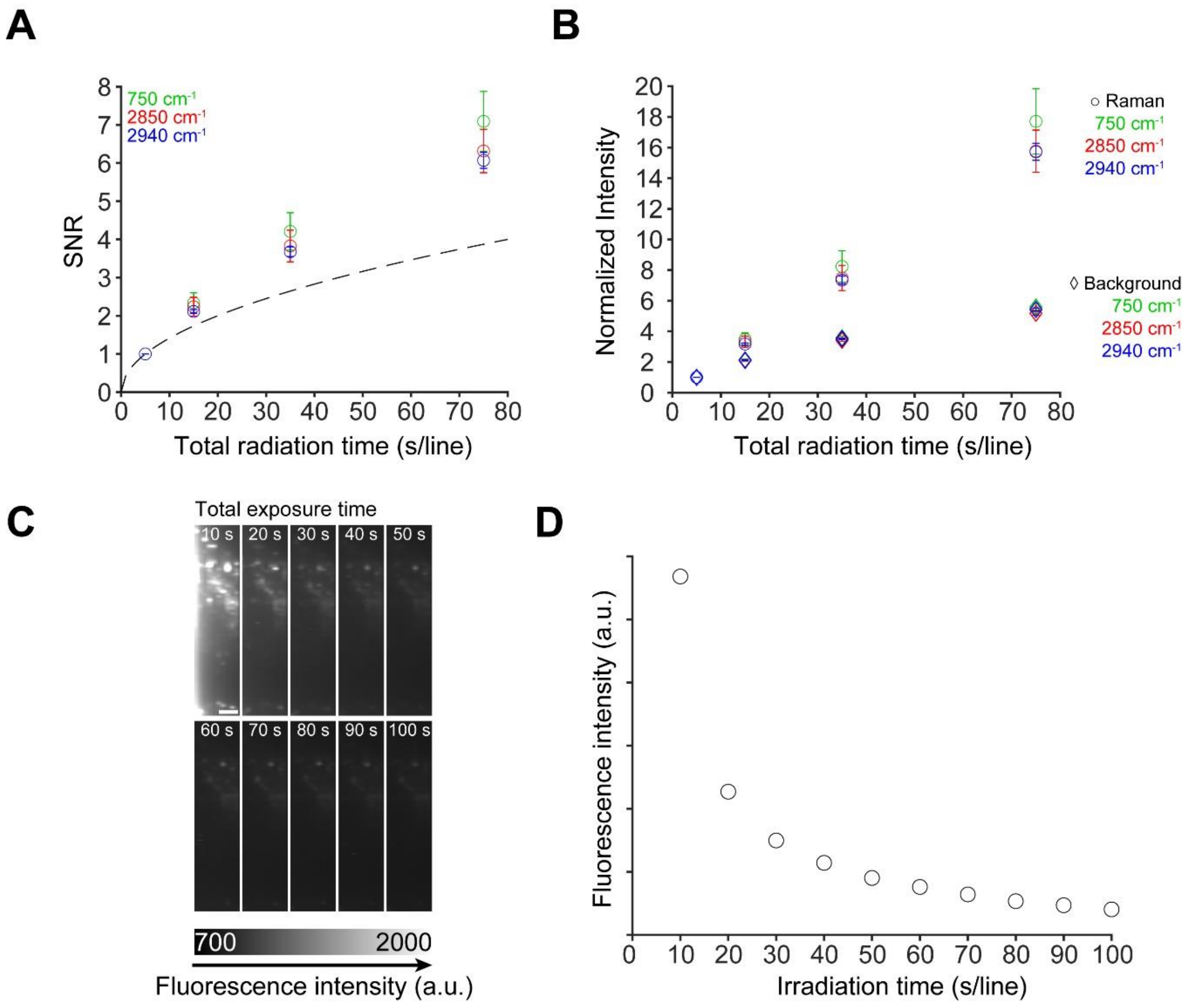

Fig. S8.

Estimated SNR in Raman spectral imaging with extended exposure times. (**A**) The SNR derived for the bands at 750 cm^{-1}, 2850 cm^{-1}, and 2933 cm^{-1} of the cryofixed HeLa cells in Fig.**2A** (circle). The SNR was normalized by the SNR value at 5 s/line for each exposure time. The normalized SNR was averaged over 12 different regions in Fig. **2A** (n = 12). The black dashed line shows the SNR for long exposures, estimated using the first data point (5 s/line). This curve theoretically assumes Poisson noise and increases along the square root of the total exposure time. However, the measured SNR is larger than the SNR theoretically estimated due to the time-dependent decrease in spectral background. (**B**) Plots of the Raman signal (the difference of the peak and bottom, circle) and the spectral background (diamond) for the same bands as in A. Increase in the spectral background intensity along total radiation time is less than linear, indicating the occurrence of photobleaching or transition to the dark state of background fluorescence. The Raman signal and spectral background intensities were averaged over 12 different regions in Fig. **2A** (n = 12). (**C**) Autofluorescence images of cryofixed HeLa cells at 203 K reconstructed by averaging the intensity between1900 and 2400 cm^{-1}. Excitation intensity was 3.0 mW/µm^2. Scale bar was 5 µm. Step size was 1.2 µm. (**D**) Irradiation time dependence of fluorescence intensity of cryofixed cells. Fluorescence intensity was averaged excluding the top and bottom 10% of each image in (C).

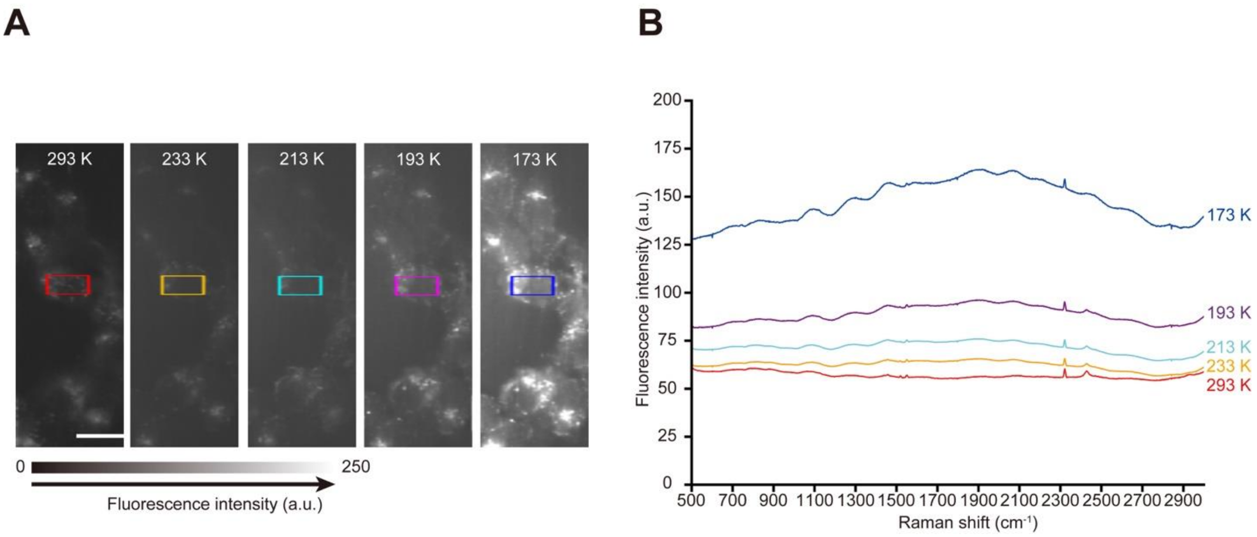

Fig. S9.

Temperature dependence of autofluorescence in Raman imaging of cells. (**A**) Raman images of HeLa cells observed repeatedly at room temperature (293 K), 233 K, 213 K, 193 K and 173 K. The images were reconstructed by the intensity at 1803-2295 cm^{-1}. (**B**) Average Raman spectra of HeLa cells. Red, orange, cyan, purple and blue lines are the spectra measured at 293 K(room temperature), 233 K, 213 K, 193 K, and 173 K, respectively. The rectangles in (A) indicates the area used to calculate the averaged Raman spectra. The exposure time, and the intensity of excitation laser were 3 s/line and 60µW/$µm^2$, respectively. The scanning step was 540 nm. Scale bar: 10 µm.

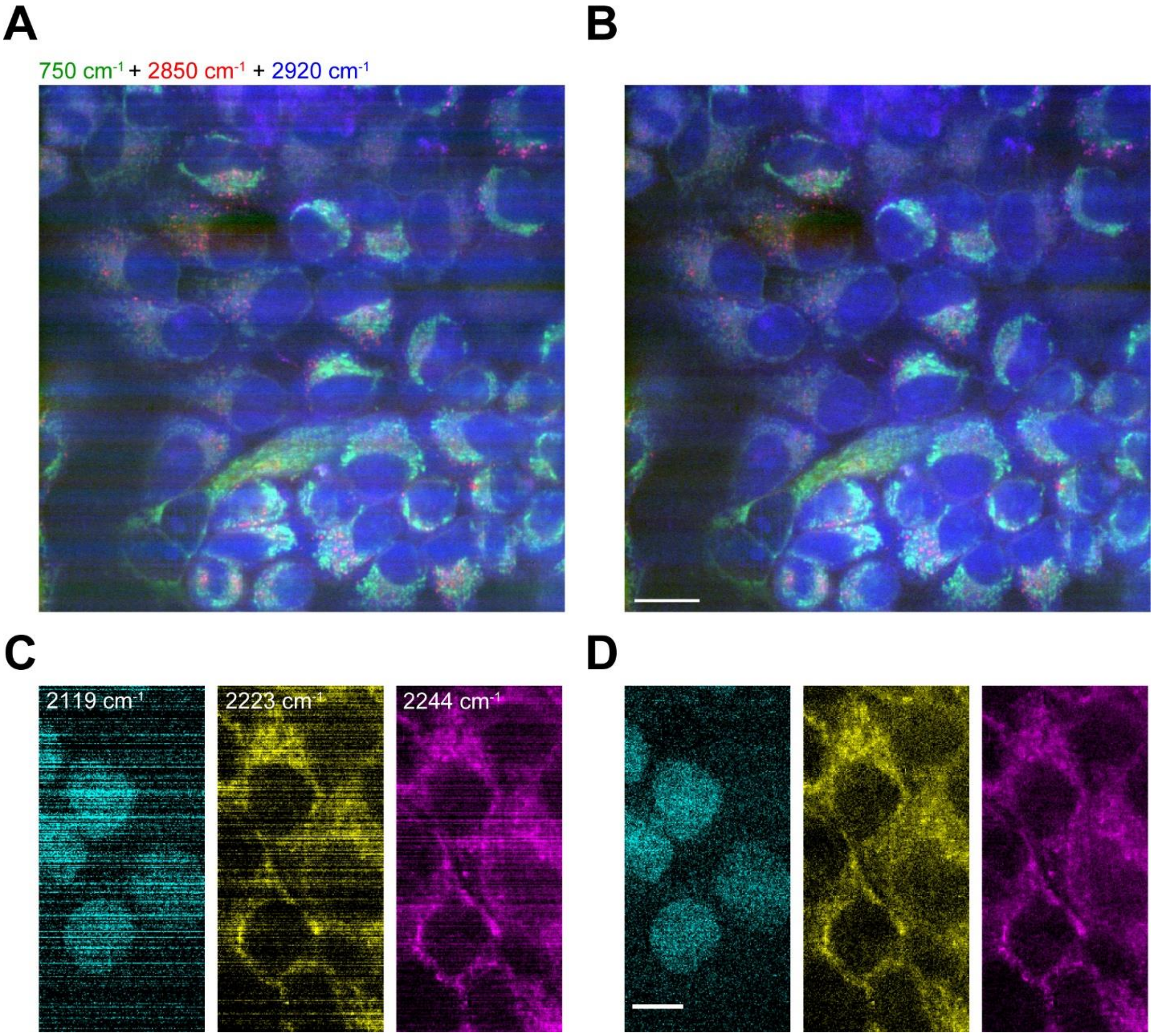

Fig. S10.

Stripe correction of Raman images. Raman images of cryofixed HeLa cells with wide FOV (**A**, **B**) and treated with multiple Raman tag (**C**, **D**), without stripe correction (**A**, **C**) and with stripe correction (**B**, **D**). All scale bars: 20 µm (**A**, **B**) and 10 µm (**C**, **D**)

Table. S1.
Parameters for the calculation of local heating.

	Sample	Absorber
Materials	Water (Ice)	FAD, Cytochrome *c*
size (x,y,z)	5 mm, 5 mm, 20 um	50 um, 200 um, 7.5 um
Absorbance @ 532 nm (cm^{-1})	0.0004 (73)	0.022
Thermal conductivity (W/mK)	2.40 (74)	2.40 (74)
Heat capacity (kJ/kgK)	1.80 (75)	1.80 (75)
Density (kg/m^3)	914 (76)	914 (76)

Table S2.

Detailed parameters for the Raman image reconstruction

	Cryo-fixed HeLa in Fig. 1 C,D	Live HeLa in Fig. 1 C,D	Live HeLa in Fig. 1 E	Cryofixed HeLa in Fig. 1 E	Cryofixed HeLa in Fig. 2 C	Cryofixed HeLa in Fig. 2 D	Live/PFA fixed HeLa in Fig. 2 D
Wavenumber range (cm^{-1})	500-3000	500-3000	700-800<br>1351-1521<br>1560-1750	700-800<br>1351-1521<br>1560-1750	500-3000<br>2000-2200	500-3000<br>2100-2315	500-3000<br>2100-2315
SVD loading for image reconstruction	1st-7th	1st-6th	1st-2nd<br>1st-2nd<br>1st-2nd	1st-4th<br>1st-6th<br>1st-3rd	1st-10th<br>1st-4th	1st-8th<br>1st-3th	1st-8th<br>1st-4th
Polynormial fitting for background subtraction	6th	6th	1st	1st	5th<br>1st	5th<br>2nd	5th<br>2nd
Band I Peak-bottom (cm^{-1})	747-726	750-730	751-736	747-735	747-727	747-726	748-729
Band II Peak-bottom (cm^{-1})	1680-1707	1680-1718	1439-1411	1437-1426	2850-2811	2244-2265	2248-2267
Band III Peak-bottom (cm^{-1})	2847-2787	2851-2805	1680-1698	1680-1707	2119-2138	N/A	N/A

	Cryofixed HeLa in Fig. 1 G	Cryofixed rat heart tissue in Fig. 3 C-D	Cryofixed HeLa in Fig. 2 A-B	Cryofixed HeLa in Fig. 2 E	Cryofixed HeLa in Fig. 2 E	Cryofixed HeLa in Fig. 2 E
Wavenumber range (cm^{-1})	500-1680<br>2780-2920	505-2010	500-3000	500-3000	2050-2315	3000-3600
Polynormial fitting for background subtraction	N/A	8th	N/A	7th	2nd	1st
SVD loading for image reconstruction	1st-21th<br>1st-10th	1st-12th	N/A	1st-14th	1st,4th,7th	1st-4th
Band I Peak-bottom (cm^{-1})	747-760	600-584	745-732	752-738	2119-2098	3128-3220
Band II Peak-bottom (cm^{-1})	2851-2810	N/A	2848-2781	795-806	2224-2203	N/A
Band III Peak-bottom (cm^{-1})	2920-2837	N/A	2934-2842	1160-1147	2244-2265	N/A
Band IV Peak-bottom (cm^{-1})	N/A	N/A	N/A	2850-2791	N/A	N/A
Band V Peak-bottom (cm^{-1})	N/A	N/A	N/A	2938-2852	N/A	N/A

REFERENCES AND NOTES

1. J.-X. Cheng, W. Min, Y. Ozeki, D. Polli, *Stimulated Raman Scattering Microscopy: Techniques and Applications*. (Elsevier, 2021).

2. Y. Kumamoto, M. Li, K. Koike, K. Fujita, Slit-scanning Raman microscopy: Instrumentation and applications for molecular imaging of cell and tissue. *J. Appl. Phys.* **132**, 171101 (2022).

3. B. G. Saar, C. W. Freudiger, J. Reichman, C. M. Stanley, G. R. Holtom, X. S. Xie. Video-rate molecular imaging in vivo with stimulated Raman scattering. *Science* **330**, 1368–1370 (2010).

4. M. Li, Y. Nawa, S. Ishida, Y. Kanda, S. Fujita, K. Fujita. Label-free chemical imaging of cytochrome P450 activity by Raman microscopy. *Commun. Biol.* **5**, 778 (2022).

5. K. Aljakouch, T. Lechtonen, H. K. Yosef, M. K. Hammoud, W. Alsaidi, C. Kötting, C. Mügge, R. Kourist, S. F. El-Mashtoly, K. Gerwert, Raman microspectroscopic evidence for the metabolism of a tyrosine kinase inhibitor, neratinib, in cancer cells. *Angew. Chem. Int. Ed. Engl.* **57**, 7250–7254 (2018).

6. Z. Farhane, F. Bonnier, A. Casey, H. J. Byrne, Raman micro spectroscopy for in vitro drug screening: Subcellular localisation and interactions of doxorubicin. *Analyst* **140**, 4212–4223 (2015).

7. E. Brauchle, A. Knopf, H. Bauer, N. Shen, S. Linder, M. G. Monaghan, K. Ellwanger, S. L. Layland, S. Y. Brucker, A. Nsair, K. Schenke-Layland, Non-invasive chamber-specific identification of cardiomyocytes in differentiating pluripotent stem cells. *Stem Cell Reports* **6**, 188–199 (2016).

8. G. Pettinato, M. F. Coughlan, X. Zhang, L. Chen, U. Khan, M. Glyavina, C. J. Sheil, P. K. Upputuri, Y. N. Zakharov, E. Vitkin, A. B. D'Assoro, R. A. Fisher, I. Itzkan, L. Zhang, L. Qiu, L. T. Perelman, Spectroscopic label-free microscopy of changes in live cell chromatin and biochemical composition in transplantable organoids. *Sci. Adv.* **7**, eabj2800 (2021).

9. K. Kong, C. Kendall, N. Stone, I. Notingher, Raman spectroscopy for medical diagnostics - From in-vitro biofluid assays to in-vivo cancer detection. *Adv. Drug Deliv. Rev.* **89**, 121–134 (2015).

10. Z. Liu, W. Su, J. Ao, M. Wang, Q. Jiang, J. He, H. Gao, S. Lei, J. Nie, X. Yan, X. Guo, P. Zhou, H. Hu, M. Ji, Instant diagnosis of gastroscopic biopsy via deep-learned single-shot femtosecond stimulated Raman histology. *Nat. Commun.* **13**, 4050 (2022).

11. J. N. Taylor, K. Mochizuki, K. Hashimoto, Y. Kumamoto, Y. Harada, K. Fujita, T. Komatsuzaki, High-resolution raman microscopic detection of follicular thyroid cancer cells with unsupervised machine learning. *J. Phys. Chem. B* **123**, 4358–4372 (2019).

12. A. Downes, A. Elfick, Raman spectroscopy and related techniques in biomedicine. *Sensors* **10**, 1871–1889 (2010).

13. Y. Tan, S. O. Konorov, H. G. Schulze, J. M. Piret, M. W. Blades, R. F. B. Turner, Comparative study using Raman microspectroscopy reveals spectral signatures of human induced pluripotent cells more closely resemble those from human embryonic stem cells than those from differentiated cells. *Analyst* **137**, 4509–4515 (2012).

14. H. Yamakoshi, K Dodo, A. Palonpon, J. Ando, K. Fujita, S. Kawata, M. Sodeoka, Alkyne-tag Raman imaging for visualization of mobile small molecules in live cells. *J. Am. Chem. Soc.* **134**, 20681–20689 (2012).

15. Y. Shen, F. Hu, W. Min, Raman imaging of small biomolecules. *Annu. Rev. Biophys.* **48**, 347–369 (2019).

16. L. Wei, Z. Chen, L. Shi, R. Long, A. V. Anzalone, L. Zhang, F. Hu, R. Yuste, V. W. Cornish, W. Min, Super-multiplex vibrational imaging. *Nature* **544**, 465–470 (2017).

17. F. Hu, C. Zeng, R. Long, Y. Miao, L. Wei, Q. Xu, W. Min, Supermultiplexed optical imaging and barcoding with engineered polyynes. *Nat. Methods* **15**, 194–200 (2018).

18. V. V. Pully, A. T. M. Lenferink, C. Otto. Time-lapse Raman imaging of single live lymphocytes. *J. Raman Spectrosc.* **42**, 167–173 (2011).

19. C. K. Huang, M. Ando, H. O. Hamaguchi, S. Shigeto. Disentangling dynamic changes of multiple cellular components during the yeast cell cycle by in vivo multivariate Raman imaging. *Anal. Chem.* **84**, 5661–5668 (2012).

20. M. Okada, N. I. Smith, A. F. Palonpon, H. Endo, S. Kawata, M. Sodeoka, K. Fujita, Label-free Raman observation of cytochrome c dynamics during apoptosis. *Proc. Natl. Acad. Sci. U.S.A.* **109**, 28–32 (2012).

21. A. J. Hobro, N. I. Smith, An evaluation of fixation methods: Spatial and compositional cellular changes observed by Raman imaging. *Vib. Spectrosc.* **91**, 31–45 (2017).

22. T. Ichikawa, D. Wang, K. Miyazawa, K. Miyata, M. Oshima, T. Fukuma, Chemical fixation creates nanoscale clusters on the cell surface by aggregating membrane proteins. *Commun. Biol.* **5**, 487 (2022).

23. M. M. Mariani, P. Lampen, J. Popp, B. R. Wood, V. Deckert, Impact of fixation on in vitro cell culture lines monitored with Raman spectroscopy. *Analyst* **134**, 1154–1161, (2009).

24. J. Huebinger, J. Spindler, K. J. Holl, B. Koos, Quantification of protein mobility and associated reshuffling of cytoplasm during chemical fixation. *Sci. Rep.* **8**, 17756 (2018).

25. L. Maneta-Peyret, P. Compère, P. Moreau, G. Goffinet, C. Cassagne, Immunocytochemistry of lipids: Chemical fixatives have dramatic effects on the preservation of tissue lipids. *Histochem. J.* **31**, 541–547 (1999).

26. P. R. Carey, J. Dong, Following ligand binding and ligand reactions in proteins via Raman crystallography. *Biochemistry* **43**, 8885–8893 (2004).

27. K. A. Okotrub, N. V. Surovtsev. Raman scattering evidence of hydrohalite formation on frozen yeast cells. *Cryobiology* **66**, 47–51 (2013).

28. T. K. Tsang, E. A. Bushong, D. Boassa, J. Hu, B. Romoli, S. Phan, D. Dulcis, C. Y. Su, M. H. Ellisman, High-quality ultrastructural preservation using cryofixation for 3D electron microscopy of genetically labeled tissues. *eLife* **7**, e35524 (2018).

29. M. H. Laporte, N. Klena, V. Hamel, P. Guichard, Visualizing the native cellular organization by coupling cryofixation with expansion microscopy (Cryo-ExM). *Nat. Methods* **19**, 216–222 (2022).

30. J. Huebinger, H. Grecco, M. E. Masip, J. Christmann, G. R. Fuhr, P. I. H. Bastiaens, Ultrarapid cryo-arrest of living cells on a microscope enables multiscale imaging of out-of-equilibrium molecular patterns. *Sci. Adv.* **7**, eabk0882 (2021).

31. T. Sugimura, S. Kajimoto, T. Nakabayashi, Label-free imaging of intracellular temperature by using the O−H stretching raman band of water. *Angew. Chem. Int. Ed. Engl.* **59**, 7755–7760 (2020).

32. K. A. Okotrub, V. I. Mokrousova, S. Y. Amstislavsky, N. V. Surovtsev, Lipid droplet phase transition in freezing cat embryos and oocytes probed by Raman spectroscopy. *Biophys. J.* **15**, 577–587 (2018).

33. H. Okamoto, Y. Sekimoto, M. Tasumi, Assignment and anharmonicity analysis of overtone and combination bands observed in the resonance Raman spectra of carotenoids. *Spectrochim. Acta A Mol. Spectrosc.* **50**, 1467–1473 (1994).

34. M. A. Welte, A. P. Gould, Lipid droplet functions beyond energy storage. *Biochim. Biophys. Acta Mol. Cell Biol. Lipids* **1862**, 1260–1272 (2017).

35. K. A. Okotrub, N. V. Surovtsev, Redox state of cytochromes in frozen yeast cells probed by resonance raman spectroscopy. *Biophys. J.* **109**, 2227–2234 (2015).

36. M. Kakita, M. Okuno, H. O. Hamaguchi, Quantitative analysis of the redox states of cytochromes in a living L929 (NCTC) cell by resonance Raman microspectroscopy. *J. Biophotonics* **6**, 256–259 (2013).

37. M. F. Nonier, N. V. De Gaulejac, N. Vivas, C. Vitry, Characterization of carotenoids and their degradation products in oak wood. Incidence on the flavour of wood. *C. R. Chim.* **7**, 689–698 (2004).

38. S. Limbo, L. Torri, L. Piergiovanni, Light-induced changes in an aqueous β-carotene system stored under halogen and fluorescent lamps, affected by two oxygen partial pressures. *J. Agric. Food Chem.* **55**, 5238–5245 (2007).

39. J. C. Gilkey, L. A. Staehelin. Advances in ultrarapid freezing for the preservation of cellular ultrastructure. *J. Electron Microsc. Tech.* **3**, 177–210 (1986).

40. J. Ma, X. Yang, Y. Sun, J. Yang, Thermal damage in three-dimensional vivo bio-tissues induced by moving heat sources in laser therapy. *Sci. Rep.* **9**, 10987 (2019).

41. S. Matsui, Y. Tsujimoto, K. Matsushima, Stimulatory effects of hydroxyl radical generation by Ga-Al-As laser irradiation on mineralization ability of human dental pulp cells. *Biol. Pharm. Bull.* **30**, 27–31 (2007).

42. K. A. K. Tanaka, K. G. N Suzuki, Y. M. Shirai, S. T. Shibutani, M. S. H. Miyahara, H. Tsuboi, M. Yahara, A. Yoshimura, S. Mayor, T. K. Fujiwara, A. Kusumi, Membrane molecules mobile even after chemical fixation. *Nat. Methods* **7**, 865–866 (2010).

43. P. Tang, W. Cheng, X. He, Q. Zhang, J. Zhong, X. Lu, S. Liu, L. Zhong, Raman spectrum spectral imaging revealing the molecular mechanism of Berberine-induced Jurkat cell apoptosis and the receptor-mediated Berberine delivery system. *Biomed. Opt. Express* **10**, 1581–1600 (2019).

44. H. Yamakoshi, A. Palonpon, K. Dodo, J. Ando, S. Kawata, K. Fujita, M. Sodeoka, A sensitive and specific Raman probe based on bisarylbutadiyne for live cell imaging of mitochondria. *Bioorg. Med. Chem. Lett.* **25**, 664–667 (2015).

45. J. Dong, J. Malsam, J. C. Bischof, A. Hubel, A. Aksan, Spatial distribution of the state of water in frozen mammalian cells. *Biophys. J.* **99**, 2453–2459 (2010).

46. H. Fujioka, M. Kawatani, S. J. Spratt, A. Komazawa, Y. Misawa, J. Shou, T. Mizuguchi, H. Kosakamoto, R. Kojima, Y. Urano, F. Obata, Y. Ozeki, M. Kamiya, Activatable Raman probes utilizing enzyme-induced aggregate formation for selective ex vivo imaging. *J. Am. Chem. Soc.* **145**, 8871–8881 (2023).

47. S. Ohira, H. Tanaka, Y. Harada, T. Minamikawa, Y. Kumamoto, S. Matoba, H. Yaku, T. Takamatsu, Label-free detection of myocardial ischemia in the perfused rat heart by spontaneous Raman spectroscopy. *Sci. Rep.* **7**, 42401 (2017).

48. N. A. Brazhe, M. Treiman, A. R. Brazhe, N. L. Find, G. V. Maksimov, O. V. Sosnovtseva, Mapping of redox state of mitochondrial cytochromes in live cardiomyocytes using Raman microspectroscopy. *PLOS ONE* **7**, e41990 (2012).

49. K. Ikemoto, K. Hashimoto, Y. Harada, Y. Kumamoto, M. Hayakawa, K. Mochizuki, K. Matsuo, K. Yashiro, H. Yaku, T. Takamatsu, H. Tanaka, Raman spectroscopic assessment of myocardial viability in langendorff-perfused ischemic rat hearts. *Acta Histochem. Cytochem.* **54**, 65–72 (2021).

50. K. A. Okotrub, N. V. Surovtsev, Effect of glycerol on photobleaching of cytochrome Raman lines in frozen yeast cells. *Eur. Biophys. J.* **47**, 655–662 (2018).

51. C. Kallepitis, M. S. Bergholt, M. M. Mazo, V. Leonardo, S. C. Skaalure, S. A. Maynard, M. M. Stevens, Quantitative volumetric Raman imaging of three dimensional cell cultures. *Nat. Commun.* **8**, 14843 (2017).

52. Y. Harada, P. Dai, Y. Yamaoka, M. Ogawa, H. Tanaka, K. Nosaka, K. Akaji, T. Takamatsu, Intracellular dynamics of topoisomerase I inhibitor, CPT-11, by slit-scanning confocal Raman microscopy. *Histochem. Cell Biol.* **132**, 39–46 (2009).

53. S. F. El-Mashtoly, D. Petersen, H. K. Yosef, A. Mosig, A. Reinacher-Schick, C. Kötting, K. Gerwert, Label-free imaging of drug distribution and metabolism in colon cancer cells by Raman microscopy. *Analyst* **139**, 1155–1161 (2014).

54. K. Koike, K. Bando, J. Ando, H. Yamakoshi, N. Terayama, K. Dodo, N. I. Smith, M. Sodeoka, K. Fujita, Quantitative drug dynamics visualized by alkyne-tagged plasmonic-enhanced Raman microscopy. *ACS Nano* **14**, 15032–15041 (2020).

55. L. Cui, K. Yang, H.-Z. Li, H. Zhang, J.-Q. Su, M. Paraskevaidi, F. L. Martin, B. Ren, Y.-G. Zhu, Functional single-cell approach to probing nitrogen-fixing bacteria in soil communities by resonance Raman spectroscopy with $^{15}N_2$ labeling. *Anal. Chem.* **90**, 5082–5089 (2018).

56. S. Vanden-Hehir, W. J. Tipping, M. Lee, V. G. Brunton, A. Williams, A. N. Hulme, Raman imaging of nanocarriers for drug delivery. *Nanomaterials* **9**, 341 (2019).

57. R. Li, K. Hornberger, J. R. Dutton, A. Hubel, Cryopreservation of human iPS cell aggregates in a DMSO-free solution—An optimization and comparative study. *Front. Bioeng. Biotechno.* **8**, 1 (2020).

58. N. Ohno, N. Terada, S. Ohno, Histochemical analyses of living mouse liver under different hemodynamic conditions by “in vivo cryotechnique”. *Histochem. Cell Biol.* **126**, 389–398 (2006).

59. N. Terada, N. Ohno, S. Saitoh, S. Ohno, Application of ‘in vivo cryotechnique’ to detect erythrocyte oxygen saturation in frozen mouse tissues with confocal Raman cryomicroscopy. *J. Struct. Biol.* **163**, 147–154 (2008).

60. K. Bando, S. Yabuuchi, M. Li, T. Kubo, R. Oketani, N. I. Smith, K. Fujita, Bessel-beam illumination Raman microscopy. *Biomed. Opt. Express* **13**, 3161–3170 (2022).

61. A. T. Lewis, R. Gaifulina, M. Isabelle, J. Dorney, M. L. Woods, G. R. Lloyd, K. Lau, M. Rodriguez-Justo, C. Kendall, N. Stone, G. M. Thomas, Mirrored stainless steel substrate provides improved signal for Raman spectroscopy of tissue and cells. *J. Raman Spectrosc.* **48**, 119–125 (2017).

62. M.-J. Anita, R.-K. Rebecca, Raman spectroscopy for the detection of cancers and precancers. *J. Biomed. Opt.* **1**, 31–70 (1995).

63. S. Chadha, W. H. Nelson, J. F. Sperry, Ultraviolet micro-Raman spectrograph for the detection of small numbers of bacterial cells. *Rev. Sci. Instrum.* **64**, 3088–3093 (1993).

64. H. Bank, Visualization of freezing damage. II. Structural alterations during warming. *Cryobiology* **10**, 157–170 (1973).

65. P. Monaghan, N. Perusinghe, M. Müller, High-pressure freezing for immunocytochemistry. *J. Microsc.* **192**, 248–258 (1998).

66. V. Kocherbitov, J. Latynis, A. Misiuì, J. Barauskas, G. Niaura. Hydration of lysozyme studied by Raman spectroscopy. *J. Phys. Chem. B* **117**, 4981–4992 (2013).

67. P. Schultz, Cryo-electron microscopy of vitrified specimens. *Q. Rev. Biophys.* **21**, 129–228 (1988).

68. R. Kaufmann, P. Schellenberger, E. Seiradake, I. M. Dobbie, E. Y. Jones, I. Davis, C. Hagen, K. Grünewald, Super-resolution microscopy using standard fluorescent proteins in intact cells under cryo-conditions. *Nano Lett.* **14**, 4171–4175 (2014).

69. D. P. Hoffman, G. Shtengel, C. S. Xu, K. R. Campbell, M. Freeman, L. Wang, D. E. Milkie, H. A. Pasolli, N. Iyer, J. A. Bogovic, D. R. Stabley, A. Shirinifard, S. Pang, D. Peale, K. Schaefer, W. Pomp, C.-L. Chang, J. Lippincott-Schwartz, T. Kirchhausen, D. J. Solecki, E. Betzig, H. F. Hess, Correlative three-dimensional super-resolution and block-face electron microscopy of whole vitreously frozen cells. *Science* **367**, eaaz5357 (2020).

70. H.-J. V. Manen, Y. M. Kraan, D. Roos, C. Otto, Intracellular chemical imaging of heme-containing enzymes involved in innate immunity using resonance Raman microscopy. *J. Phys. Chem. B* **108**, 18762–18771 (2004).

71. C. A. Lieber, A. Mahadevan-Jansen, Automated method for subtraction of fluorescence from biological raman spectra. *Appl. Spectrosc.* **57**, 1363–1367 (2003).

72. K. Mochizuki, Y. Kumamoto, S. Maeda, M. Tanuma, A. Kasai, M. Takemura, Y. Harada, H. Hashimoto, H. Tanaka, N. I. Smith, K. Fujita, High-throughput line-illumination Raman microscopy with multislit detection. *Biomed. Opt. Express* **14**, 1015–1026 (2023).

73. R. M. Pope, E. S. Fry, Absorption spectrum (380–700 nm) of pure water. II. Integrating cavity measurements. *Appl. Opt.* **36**, 8710–8723 (1997).

74. O. Haida, T. Matsuo, H. Suga, S. Seki, Calorimetric study of the glassy state X. Enthalpy relaxation at the glass-transition temperature of hexagonal ice. *J. Chem. Thermodynamics* **6**, 815–825 (1974).

75. G. A. Slack Thermal conductivity of ice. *Phys. Rev. B.* **22** 3065–3071 (1980).

76. K.-H. Liu, Y. Zhang, J.-J. Lee, C.-C. Chen, Y.-Q. Yeh, S.-H. Chen, C.-Y. Mou, Density and anomalous thermal expansion of deeply cooled water confined in mesoporous silica investigated by synchrotron X-ray diffraction. *J. Chem. Phys.* **139**, 064502 (2013).
